# Dual Regulation of the Small RNA MicC and the Quiescent Porin OmpN in Response to Antibiotic Stress in *Escherichia coli*

**DOI:** 10.3390/antibiotics6040033

**Published:** 2017-12-06

**Authors:** Sushovan Dam, Jean-Marie Pagès, Muriel Masi

**Affiliations:** UMR_MD1, Aix-Marseille Univ & Institut de Recherche Biomédicale des Armées, 27 Boulevard Jean Moulin, 13005 Marseille, France; sushovan.dam@etu.univ-amu.fr (S.D.); jean-marie.pages@univ-amu.fr (J.-M.P.)

**Keywords:** *Escherichia coli*, outer membrane porins, regulatory small RNAs, membrane transport, antibiotic susceptibility

## Abstract

Antibiotic resistant Gram-negative bacteria are a serious threat for public health. The permeation of antibiotics through their outer membrane is largely dependent on porin, changes in which cause reduced drug uptake and efficacy. *Escherichia coli* produces two major porins, OmpF and OmpC. MicF and MicC are small non-coding RNAs (sRNAs) that modulate the expression of OmpF and OmpC, respectively. In this work, we investigated factors that lead to increased production of MicC. *micC* promoter region was fused to *lacZ*, and the reporter plasmid was transformed into *E. coli* MC4100 and derivative mutants. The response of *micC–lacZ* to antimicrobials was measured during growth over a 6 h time period. The data showed that the expression of *micC* was increased in the presence of β-lactam antibiotics and in an *rpoE* depleted mutant. Interestingly, the same conditions enhanced the activity of an *ompN–lacZ* fusion, suggesting a dual transcriptional regulation of *micC* and the quiescent adjacent *ompN*. Increased levels of OmpN in the presence of sub-inhibitory concentrations of chemicals could not be confirmed by Western blot analysis, except when analyzed in the absence of the sigma factor σ^E^. We suggest that the MicC sRNA acts together with the σ^E^ envelope stress response pathway to control the OmpC/N levels in response to β-lactam antibiotics.

## 1. Introduction

Antibacterial resistance is broadly recognized as a growing threat for human health [1,2,3]. As such, increasing antibiotic treatment failures due to multidrug resistant (MDR) bacteria have stirred the urgent need to better understand the underlying molecular mechanisms and promote innovation, with the development of new antibiotics and alternative therapies [4,5]. The efficacy of antibacterial compounds depends on their capacity to reach inhibitory concentrations at the vicinity of their target. This is particularly challenging for drugs directed against Gram-negative bacteria, which exhibit a complex envelope comprising two membranes and transmembrane efflux pumps [6]. The Gram-negative envelope comprises an inner membrane (IM), which is a symmetric phospholipid bilayer; a thin peptidoglycan (PG) layer ensuring the cell shape; and an outer membrane (OM) that is an asymmetric bilayer, composed of an inner phospholipid leaflet and an outer leaflet of lipopolysaccharide (LPS) [7]. First, the OM is a barrier to both hydrophobic and hydrophilic compounds, including necessary nutrients, metabolic substrates and antibiotics, but access is provided by the water filled β-barrel channels called porins [8,9]. In *Escherichia coli*, the channels of the general porins OmpF and OmpC, are size restricted, and show a preference for passage of hydrophilic charged compounds, including antibiotics such as β-lactams and fluoroquinolones. Second, constitutive tripartite RND (resistance–nodulation–cell division) efflux pumps, such as the AcrAB–TolC pump of *E. coli*, play a major role in removing antibiotics from the periplasm [10]. Importantly, it has been noted that the efflux pumps are synergized by the OM, since, once ejected into the extracellular space, compounds must re-traverse the restricted-permeability OM barrier [10]. Not surprisingly, MDR clinical isolates of *Enterobacteriaceae* generally exhibit porin loss and/or increased efflux, which both contribute to reduce the intracellular accumulation of antibiotics below the threshold that would be efficient for activity [9,10,11].

Given the importance of the OM in controlling the uptake of beneficial as well as toxic compounds, one can expect that the expression of porins depends on environmental factors, and is well-coordinated at the transcriptional and post-transcriptional levels. Best studied transcriptional regulators are the IM sensor kinase EnvZ and its cognate response regulator OmpR [12]. EnvZ autophosphorylates in response to a specific envelope stress, such as high osmolarity, then transfers its phosphate group to OmpR. OmpR and OmpR-P have different binding affinities to the porin promoters. At low osmolarity, OmpR activates *ompF* transcription, whereas at high osmolarity, OmpR-P represses *ompF* transcription and activates *ompC* transcription. This differential regulation of OmpF and OmpC is consistent with that in high osmolarity environments, such as in a host where nutrients are abundant, the small pore porin OmpC is predominant, thus limiting the uptake of toxic bile salts; whereas in low osmolarity environments where nutrients are scarce, the large pore porin OmpF is expressed [8]. EnvZ–OmpR [12] and CpxA–CpxR [13] are the main two-component systems involved in the transcriptional control of OmpF and OmpC. Interestingly, the two systems are interconnected [14], and mutations have been found in response to antibiotic stresses [15] (Masi M, Pagès J.-M and Kohler T, personal observations).

The post-transcriptional repression of OmpF by the small regulatory RNA (sRNA) MicF has been discovered in 1984 [16,17,18]. This 93 nucleotide (nt) RNA is divergent to the *ompC* gene, and acts by direct base-pairing to a region that encompasses the ribosome binding site (RBS) and the start codon of the *ompF* mRNA, thus preventing translation initiation [19]. The expression of the MicF sRNA is subjected to multiple signals and regulatory pathways [20]. Positive regulation includes EnvZ–OmpR in high osmolarity conditions [21], SoxS in response to oxidative stress [22], and MarA in response to antibiotic stress [23]. The 109 nt MicC sRNA has been discovered more recently, and shown to repress OmpC by direct base-pairing to a 5′ untranslated region of the *ompC* mRNA [24]. Interestingly, MicC is transcribed clockwise, and is opposite to the adjacent *ompN* gene that encodes a quiescent porin homologous to OmpF and OmpC [25]. Due to the similar genetic organization of *ompN–micC* and *ompC–micF*, and the co-induction of *ompC* and *micF* under specific conditions (i.e., high osmolarity via EnvZ–OmpR), it has been suggested that *ompN* and *micC* could also be subjected to dual regulation [24]. With the recent interest in post-transcriptional regulators, additional sRNAs that modulate expression of abundant OM proteins have been found. As yet, the *ompC* mRNA is targeted by multiple sRNAs MicC [24], RybB [26], RseX [27], and IpeX [28,29,30]. To date, external growth conditions and regulatory factors that control the expression of MicC and/or OmpN remain largely unknown.

In this work, we first examined the transcription of *micC* and *ompN* in *E. coli* MC4100 cells grown under a series of external conditions by using *lacZ* transcriptional fusions and β-galactosidase assays. We optimized the assay by using 96-well microtiter plates, and screened the entire collection of compounds provided by the Biolog Phenotype MicroArrays^TM^ for bacterial chemical susceptibility, in order to extend the range of putative inducing cues. Results showed that high concentrations of carbapenems and cephalosporins, two clinically relevant classes of β-lactams, induce both *micC* and *ompN*. Then, the impact of carefully chosen inducing conditions on the expression levels of OmpC and OmpN was tested by Western blotting with appropriate antisera. Because the OmpN protein was undetectable in the presence of mild antibiotic stress conditions, we reasoned to investigate the transcription of *micC* and *ompN* in a series of MC4100 derivatives carrying null mutations or multicopy plasmids in order to identify putative transcriptional regulators. Interestingly, we found that OmpN was specifically expressed when the envelope stress sigma factor σ^E^ was depleted by the overexpression of the anti-sigma RseA, or when the *hns* gene encoding the histone nucleoid structuring protein, H-NS, was inactivated. Finally, we examined the functional relevance of OmpN as compared to OmpC and OmpF, with respect to drug translocation.

All these data are discussed considering the current knowledge on the Gram-negative envelope stress response pathways.

## 2. Results

### 2.1. Screening of MicC and OmpN Inducing Conditions Using LacZ Transcriptional Fusions and Biolog^TM^ Plates

**Figure 1:**
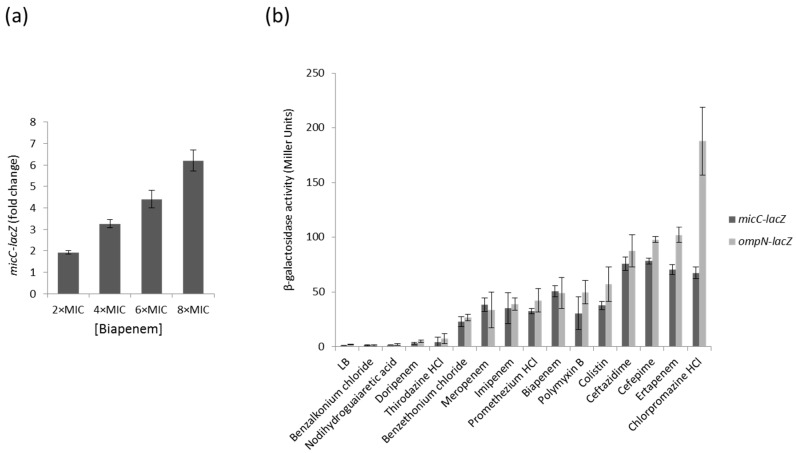
(**a**) Dose dependent *micC–lacZ* activity in presence of increasing concentrations of biapenem (MIC of 0.32 µg/mL); (**b**) β-galactosidase activity of the *micC-* and *ompN-lacZ* fusions in the presence of selected compounds. Values are means from three independent determinations, and standard deviation is represented.

Changes in porin expression play a major role in the development of antibacterial resistance. Because increased levels of MicC are associated with a decreased expression of OmpC, we aimed to examine the expression profile of the MicC sRNA by using a *micC–lacZ* transcriptional fusion in MC4100 cells grown under a series of growth conditions and β-galactosidase assays. First, we selected a number of representative growth conditions, some of which are sensed by known regulatory factors: growth phase (stationary phase accumulates RpoS), exposure to heat shock, high osmolarity (activates EnvZ–OmpR), iron or nitrogen starvation, or exposure to chemicals, such as salicylate (activates MarA), paraquat (activates SoxR/S), or different classes of antibiotics (β-lactams and fluoroquinolones). To determine whether MicC and OmpN are co-regulated, the β-galactosidase activity of an *ompN–lacZ* transcriptional fusion was also tested in MC4100 grown under the same conditions. These preliminary assays showed that growth conditions that are known to induce specific regulatory factors, such as RpoS, EnvZ–OmpR, MarA and SoxR/S, do not significantly affect the activity of the *micC–* and *ompN–lacZ* fusions, suggesting that the expression of MicC and OmpN is not controlled by these regulators. Instead, these assays allowed the identification of β-lactams potent inducers of both the *micC–* and *ompN–lacZ* fusions. As an example, Figure 1a shows that increasing concentrations of the carbapenem biapenem were accompanied with increased β-galactosidase activities. In order to extend the range of putative inducing compounds, we optimized the β-galactosidase assay using preloaded 96-well microtiter plates, and then screened Phenotype MicroArrays^TM^ plates (Biolog PM11 to PM19) for bacterial chemical susceptibility (Supplementary Data 1). A total of 18 compounds were found to increase the activity of the *micC–* and *ompN–lacZ* fusions more than 10 times, and 6 of them were selected for further investigations. Concentrations of compounds for β-galactosidase assays adapted to microtiter plates were determined with respect to their MICs (Supplementary Data 2). The data showed that the activity of the *micC–* and *ompN–lacZ* fusions were strongly increased when cells were exposed to carbapenems (i.e., biapenem and ertapenem) or cephalosporins (i.e., ceftazidime and cefepime) (Figure 1b). Interestingly, these compounds belong to the most potent subclasses of clinically used β-lactams used for treating Gram-negative infections. Other strong inducers include antiseptics (e.g., benzalkonium chloride and benzethonium chloride) and anesthetics (e.g., chlorpromazine HCl), which are also used in the clinics (Figure 1b).

### 2.2. Effects of MicC and OmpN Inducing Conditions on the Expression Levels of OmpC and OmpN

**Figure 2:**
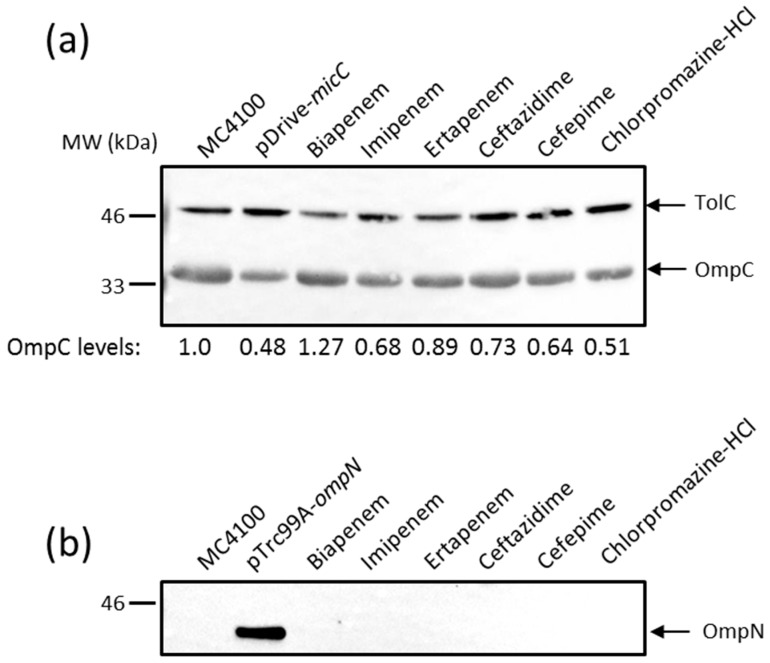
Western blot (WB) analysis of outer membrane (OM) proteins. Cells were grown, and OM extracts were prepared as described in the Materials and Methods. OM proteins equivalent to 0.2 OD_600_ units of cultures were separated by SDS-PAGE, electrotransferred on nitrocellulose membranes, and blotted with the appropriate anti-sera. Data show the production of OmpC (**a**) and OmpN (**b**). Both the positive controls pDrive-*micC* and pTrc99A-*ompN* were induced by 0.4 mM IPTG for 3 h. TolC expression was used for normalizing sample loading, and the expression of normalized OmpC has been expressed in mean values from three independent experiments.

The effect of MicC overexpression on *ompC* expression was first examined by monitoring OmpC protein levels directly. MC4100 was transformed with the MicC overexpression plasmid (pSD01) and the corresponding empty vector (pDrive). Cultures were induced with IPTG to allow MicC expression, OM extracts were prepared, and levels of OmpC were analyzed by Western blot (WB) with specific anti-peptide antibodies. As shown in Figure 2a, the overexpression of MicC clearly resulted in reduced OmpC levels, confirming that the MicC sRNA represses *ompC* expression. As noted in the section above, high *micC–lacZ* activities were obtained in the presence of high concentrations of compounds, which were detrimental for the cell growth. Therefore, MC4100 was cultured in the presence of sub-inhibitory concentrations of inducing compounds—namely biapenem, imipenem, ertapenem, ceftazidime, cefepime, and chlorpromazine HCl—in order to obtain exponentially grown cells and examine their effect on OmpC protein levels. As shown in Figure 2a, these conditions only weakly altered OmpC levels.

Given the co-induction of *micC* and *ompN*, we also tested whether OmpN expression was increased in the same samples. As a control, MC4100 was transformed with the OmpN overexpression plasmid (pSD04) and the corresponding empty vector (pTrc99A). Cultures were induced with IPTG to allow OmpN expression; OM extracts were prepared and tested for OmpN expression by WB. For this, we generated antibodies against a peptide in loop 7 present in OmpN, but absent in OmpF and OmpC. A single protein of about 39 kDa was detected in the OM extracts of MC4100 (pSD04), but not in that of MC4100 (pTrc99A), suggesting that the detected band is OmpN without cross-reactivity to other porins, and that OmpN production from the chromosome is undetectable (Figure 2b). However, OmpN production was also undetectable in OM extracts prepared from cells grown in the presence of sub-inhibitory concentrations of *micC* inducing compounds (Figure 2b).

These results suggest that transient exposure of the cells to sub-inhibitory concentrations of *micC–lacZ* inducing compounds was not sufficient to yield high levels of MicC and concomitant changes in the porin expression profile. Moreover, it is worth to note that Western blot analysis only provides steady-state levels of OmpC and OmpN. Additional reverse transcription PCR and pulse-chase experiments are needed to conclude the effects of *micC* and *ompN* inducing conditions on the expression of OmpC and OmpN at the transcriptional and post-transcriptional levels, respectively.

### 2.3. Identification of Genetic Factors That Impact on MicC and OmpN Expression

*micC–lacZ* and *ompN–lacZ* transcriptional fusions were transformed into MC4100 derivatives carrying either chromosomal null mutations or overexpression plasmids of several regulatory factors, in order to identify putative repressors or activators, respectively. In *Enterobacteriaceae*, global regulators MarA and RamA have been reported to induce MDR associated with an increase in efflux pump production and a decrease in OmpF expression levels [31,32]. We detected no induction of the reporter fusions, either when these factors were overexpressed from multicopy plasmids or when the corresponding genes were inactivated (data not shown). This observation suggests that the *micC–ompN* operon is not part of the MarA and RamA regulatory pathways, or is strongly silenced by an upstream repressor.

**Figure 3:**
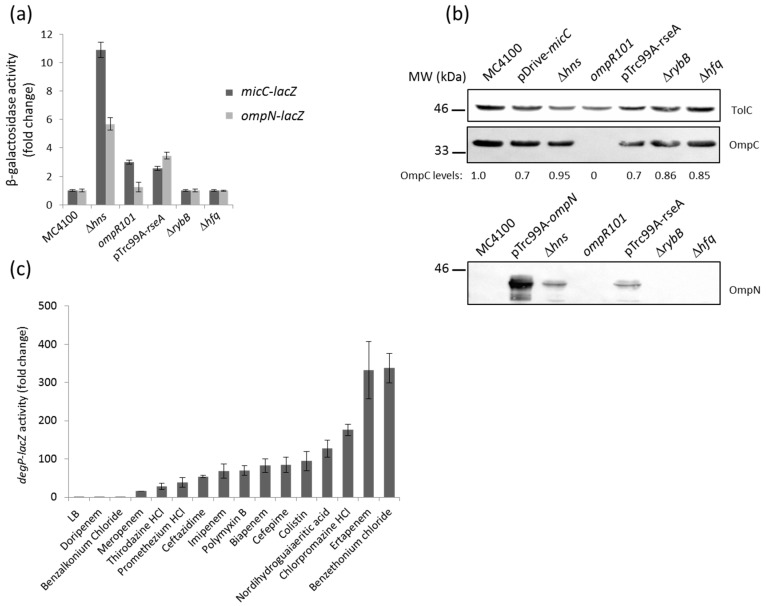
(**a**) β-Galactosidase activity of the *micC–* and *ompN–lacZ* fusions in different genetic backgrounds. Envelope stress sigma factor σ^E^ is essential in *Escherichia coli*. Therefore, cells were temporarily depleted of σ^E^ by the overexpression of the anti-sigma factor RseA with 0.4 mM IPTG under heat shock conditions at 42 °C; (**b**) WB analysis of OM proteins. Cells were grown, and OM extracts were prepared as described in the Materials and Methods. OM proteins equivalent to 0.2 ODU of cultures were separated by SDS-PAGE, electrotransferred on nitrocellulose membranes, and blotted with the appropriate anti-sera. Data show the production of OmpC (upper panel) and OmpN (lower panel). TolC expression was evaluated for normalizing sample loading and the expression of normalized OmpC has been expressed in numerical values below the bands; (**c**) β-galactosidase activity of a *degP–lacZ* chromosomal fusion in response to various external stresses.

Previous Northern blotting analysis showed that the expression of MicF (repressor of OmpF) was opposite to that of MicC (repressor of OmpC) under most of the tested conditions [24]. Because the osmoregulator OmpR is known to modulate MicF and control the opposite expression of OmpF and OmpC, we tested the impact of an *ompR* mutation on *micC* and *ompN* expression. Here, the activity of the *micC–lacZ*, but not that of the *ompN-lacZ* fusion, was slightly increased in the *ompR* null mutant, thus confirming that OmpR represses MicC (Figure 3a). Whether this regulation is direct or indirect is still unknown.

The last decade has been marked by the identification of several sRNAs. These are differentially expressed, and have been assigned to various important regulons of *E. coli* and *Salmonella*. Examples include the RyhB sRNA as a member of the iron-responsive Fur regulon [33]; MicA and RybB, which are activated by the envelope stress sigma factor, σ^E^ [26,34,35]; CyaR, whose transcription is governed by the cAMP-CRP complex [36,37]; ArcZ and FnrS, which respond to oxygen availability via the ArcA/B or Fnr systems [38,39]; MgrR, which is a member of the Mg^2+^-responsive PhoP/Q regulon [40]; SdsR, which is selectively transcribed by the major stationary phase and stress sigma factor, σ^S^ [41]; and CpxQ, which responds to the CpxA/R two-component envelope stress system [42,43]. Focusing on envelope stress responses and expression of OM proteins, we examined the impact of CpxA/R and σ^E^ on *micC* and *ompN* induction. Constitutive activation of the Cpx stress response, by multicopy plasmids expressing an autoactivated CpxA [15] or the signaling lipoprotein NlpE [44], did not increase the activity of the reporter fusion (data not shown). In the opposing scenario, when cells were depleted of σ^E^ upon the overexpression of its cognate anti-sigma RseA, the activity of both the *micC–* and *ompN–lacZ* fusions resulted in a 3–4-fold increase (Figure 3a). Additionally, OmpN was detected in OM extracts of cells grown under the same conditions (Figure 3b). We suggest this regulation is most likely indirect, as the *micC–ompN* intergenic region does not contain a σ^E^ core promoter motif [45]. Because RybB is one of the most abundant sRNA, represses OmpC as well as other OM proteins, and is part of the σ^E^ regulon in *E. coli* [46], we hypothesized that OmpN could be silenced by RybB. However, the activity of the *ompN–lacZ* fusion did not increase in *rybB* and *hfq* mutants, suggesting that the *ompN* mRNA is not targeted by RybB or any other Hfq-dependent sRNA (Figure 3a,b).

In order to explore the connection between σ^E^ and the MicC/OmpN inducing compounds, we examined the effect of the latter on the expression of DegP, a periplasmic protease/chaperone member of the σ^E^ regulon, by using a *degP–lacZ* fusion [47]. Interestingly, all the compounds that had been identified as inducers of *micC–* and *ompN–lacZ* also activated *degP–lacZ* (Figure 3c). These results suggest a strong link between toxic compounds that target the bacterial envelope, the envelope stress σ^E^ pathway, and MicC/OmpN expression [48].

Previous studies on porin regulation reported that the H-NS nucleoid protein binds to the *micF–ompC* intergenic region. Expression of the major OM proteins, OmpF and OmpC, is affected by *hns* mutations, such that OmpC expression increases via direct effect at the transcriptional level, while OmpF expression decreases via indirect regulation by the MicF sRNA at the post-transcriptional level [49,50]. Comparative transcriptomic and proteomic studies further confirmed the influence of H-NS on the expression of OmpF and OmpC, but also indicated that *ompN* was upregulated in an *hns* mutant [51]. Here, the activity of both the *micC–* and *ompN–lacZ* fusions was significantly increased (approximately by 11- and 6-fold, respectively) in an *hns* mutant (Figure 3a). The OM profile of this mutant is shown and indicates that the expression level of both OmpC and OmpN is increased by 2–3-fold (Figure 3b). Considering that MicC functions as a repressor of OmpC, negative regulation of non-identified OmpC repressors by H-NS could explain upregulation of OmpC in the *hns* mutant.

### 2.4. Role of OmpN in Antibiotic Translocation

**Figure 4:**
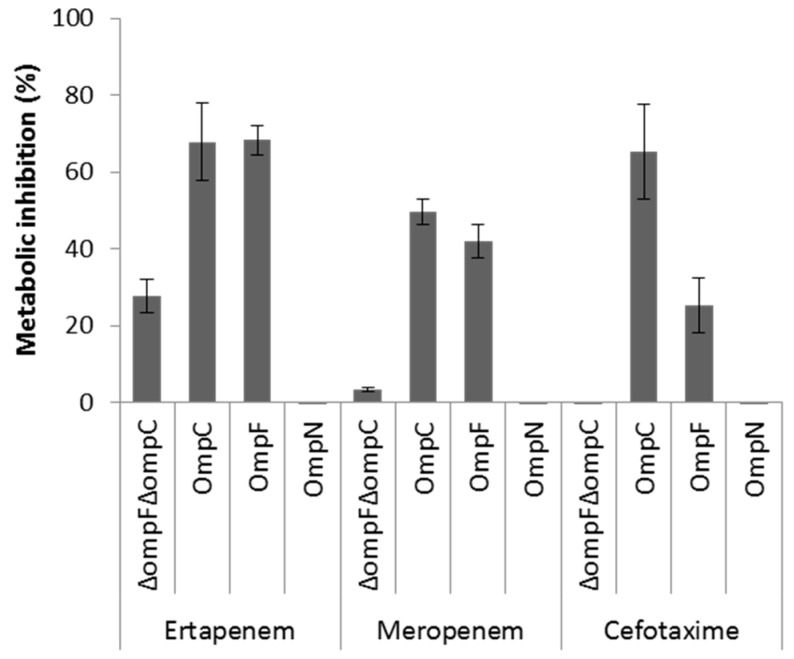
Metabolic inhibition of intact cells expressing OmpF, OmpC, or OmpN in the presence of selected β-lactam antibiotics using a resazurin-reduction-based assay. Actively metabolizing bacterial cells are able to reduce blue resazurin into red resofurin, which emits fluorescence at 590 nm. The experiment was performed in a microtiter plate, and fluorescence was measured every 10 min with an excitation wavelength of 530 nm and an emission wavelength of 590 nm. Inhibition of resazurin reduction in the presence of appropriate concentrations of each antibiotic was translated into % metabolic inhibition.

OmpF and OmpC porins represent the preferred route for the uptake of β-lactam antibiotics across the OM of *E. coli* [6,8,9]. Although OmpN is quiescent porin in *E. coli* [25], the orthologous OmpK37 of *Klebsiella pneumoniae* has been shown to be expressed at low levels under standard laboratory growth conditions, but highly expressed in β-lactam-resistant clinical isolates [52]. As a first step to investigate the role of MicC/OmpN in antibiotic susceptibility profile, we examined the expression levels of OmpF, OmpC, and OmpN in a collection of *E. coli* β-lactam-resistant clinical isolates by WB analysis. None of these isolates produced detectable OmpF, OmpC, or OmpN (Supplementary Data 3). Here, it should be noted that the anti-OmpN antibodies are directed against amino acid residues of the extracellular loop 7, which are specific of *E. coli* OmpN, but also submitted to variability between strains of this species. The impact of MicC in the downregulation of OmpC in these isolates is not known, and should be further investigated by Northern blot analysis. Second, we used a whole cell-based assay to compare the role of OmpN to that of OmpF and OmpC in the uptake of β-lactam antibiotics. To do this, the metabolic activity of *E. coli* W3100Δ*ompF*(pTrc99A) (OmpF^−^ OmpC^+^), W3100Δ*ompC*(pTrc99A) (OmpF^+^ OmpC^−^), W3100Δ*ompF*Δ*ompC*(pTrc99A) (OmpF^−^ OmpC^−^) and W3100Δ*ompF*Δ*ompC*(pSD04) (OmpF^+^ OmpC^−^ OmpN^+^) was monitored in the absence and in presence of representative β-lactams added at inhibitory concentrations, with regards to their capacity to inhibit the reduction of the viability dye resazurin [6]. The results showed that the metabolic activity of *E. coli* expressing either OmpF or OmpC, but not OmpN, was significantly inhibited upon exposure to β-lactams, suggesting that OmpN is not competent for translocation of this class of antibiotics (Figure 4). However, other approaches, such as liposome swelling assays with reconstituted OmpN, are necessary to conclude on this point.

OmpF and OmpC channels are also used for the translocation of various colicins across the OM of *E. coli* [53]. We examined the sensitivity of *E. coli* strains expressing OmpF, OmpC, or OmpN to colicins E2 and E3, by spotting serial 2-fold dilutions onto cell lawns. Interestingly, the expression of any of the three porins yields similar sensitivity (titers of 2 × 10^−7^), suggesting that OmpN channels are able to bind and transport porin-dependent group A colicins across the OM of *E. coli* (data not shown). This also points to the different mechanism of antibiotic versus colicin translocation through OM porin channels.

## 3. Discussion

sRNAs have become important players in bacterial gene regulation. To date, systematic genome-wide searches have led to the identification of approximately 80 sRNAs in *E. coli*, the majority of which are conserved in *Salmonella* and other closely related species. About one-third of the reported sRNAs repress synthesis of OM proteins. Evidence for important roles of sRNAs in this post-transcriptional regulation was previously established by the fact that the loss of Hfq, the sRNA chaperone [54], results in the overproduction of OM proteins [24,26,27,36,37,41].

In *E. coli*, the conserved Hfq-associated sRNA, MicC, was identified as a repressor of the synthesis of OmpC [24,54]. MicC inhibits the 30S ribosome binding through a conserved 22 bp RNA duplex near the start codon of the *ompC* mRNA [24]. Many parallels have been drawn between the MicC and MicF sRNAs. Both repress the expression of abundant porins by base pairing near the RBS, thereby blocking translation. Both are encoded opposite to another porin gene. Both are also conserved, together with their *omp* target sequences in *Salmonella*, *K. pneumoniae*, and *Enterobacter* spp. However, major questions such as (i) environmental conditions and/or intracellular regulatory pathways that promote maximal expression of MicC; (ii) the co-regulation of MicC and OmpN; (iii) the impact of such regulation on antibiotic susceptibility; and (iv) the prevalence of MicC/OmpN in MDR clinical isolates remain unanswered. In this work, we used *lacZ* transcriptional fusions and β-galactosidase assays to show that the expression of *micC* and *ompN* is co-regulated in response to antibiotic stress. In particular, β-lactam antibiotics are among the most potent inducers of both *micC* and *ompN*. Interestingly, we found that expression of OmpN from a plasmid could not restore the susceptibility of an *E. coli* porin-less strain to β-lactams. In addition, other studies have demonstrated that strains expressing OmpN, but not OmpF or OmpC, were less susceptible to β-lactams [52,55].

Our results also identified that envelop stress sigma factor σ^E^ and H-NS are two major negative regulators of MicC/OmpN. σ^E^ is widespread among pathogenic and non-pathogenic bacteria, and becomes activated when bacterial envelope homeostasis is perturbed due to misfolding of OM proteins in the periplasm, or severe OM damage by external stresses [56]. In both cases, the bacteria must decrease the synthesis of major OM proteins. It has been shown that MicA and RybB are the two most abundant sRNAs responsible for the rapid decay of *omp* mRNAs upon activation of the σ^E^ envelope stress response [46,57]. Although β-lactams were found to be potent inducers of the σ^E^ envelope stress response, RybB nor any other Hfq-dependent sRNA could be responsible for *ompN* silencing. This suggests that *ompN* is not subjected to sRNA post-transcriptional regulation. On the other hand, H-NS is a major component of the bacterial nucleoid, and has pleiotropic effects on gene expression, genome stability, and DNA recombination. Previous work has shown that H-NS was required for full expression of OmpF, and that this involves a role for H-NS in repressing the expression of MicF sRNA [48]. Our results also showed that H-NS had a role in repressing the expression of MicC and OmpN.

## 4. Materials and Methods

### 4.1. Plasmids and Bacterial Strains

All the *E. coli* strains and plasmids used in this study are listed in Table 1. *E. coli* MC4100 and derivatives were used for *lacZ* reporter gene assays and protein expression analysis. Knockout mutants were generated by P1 transduction from different sources and cured by using the FLP helper plasmid pCP20 to remove the kanamycin resistance cassette [58]. Strains were routinely grown in Luria Bertani (LB) broth (Sigma, Saint Quentin Fallavier, France), supplemented with the following antibiotics when necessary: ampicillin, 100 μg/mL (Amp); kanamycin, 50 μg/mL (Kan); chloramphenicol (Cam), 30 μg/mL; streptomycin 50 μg/mL (Str). *E. coli* W3110 and derivatives were used for translocation assays.

### 4.2. Plasmid Construction

Genomic DNA was extracted from MC4100 by using the Wizard^®^ Genomic purification kit (Promega, Charbonnières-les-Bains, France) according to the manufacturer’s instructions, and used as a template for all PCR-amplifications. *micC–* and *ompN–lacZ* transcriptional fusions were constructed in the promoter-less *lacZ* containing vector pFus2K [59]. A 184 nt fragment containing the MicC promoter was amplified by using the primer pair SD1 (5′-*TTACGTATC*GGATCCTCGGGGAGTGAAAACATCCT-3′) and SD2 (5′-GCGGATCCCCGCGCAGAATAACGTAT-3′), which contain BamHI restriction sites (underlined) for classic restriction/ligation cloning into BamHI restricted pFus2K (Supplementary data 4) in the orientation of *micC–lacZ* (pSD02). Because the transcription start of *ompN* is only based on promoter prediction, the entire intergenic region between MicC and OmpN was PCR-amplified by using the primer pair SD3 (5′-*GAGCTCGCATGC*GGATCCTGAATAAATCCTTTAGTTATT-3′) and SD4 (5′-*CAGGACTCTAGA*GGATCCCCGCGCAGAATAACGTAT-3′). This generated a 227 nt fragment, which contained BamHI restriction sites (underlined) and extension homologous to BamHI restricted pFus2K for cloning using the In-Fusion ™ cloning kit (Clontech, Saint Germaine n Laye, France), in the orientation of *ompN–lacZ* (pSD03) (Supplementary Data 4). For overexpression of the MicC sRNA, a 410 nt PCR fragment was generated by using the primer pair SD1 and SD5 (5′-*AGGCTCGAG*AAGCTT AGATGCTGCAGCTGAATTTG-3′) inserted into the pDrive vector restricted with BamHI and HindIII under the control of an IPTG inducible promoter by using the In-Fusion ™ cloning kit (pSD01) (Supplementary Data 1). Recombinant plasmids pSD04 and pSD05 were obtained by InFusion cloning of fragments into the pTrc99A vector after digestion with appropriate restriction enzymes. pSD04 contains ompN, which was PCR-amplified by using the primer set SD6 (5′-*CATG*GAATTCATGAAAAGCAAAGTACTGGCAC-3′) and SD7 (5′-*CGACTCAGA*GGATCCTTAGAACTGATAAACCAGACCTAAAGCG-3′) that contain the EcoRI and BamHI restriction sites respectively. pSD05 contains rseA, which was PCR-amplified by using the primer pair SD8 (5′-*GGTATTAG*CCATGGAGAAAG-3′) and SD9 (5′-*CTGTGCCGC*CCCGGGTACTTTCTG-3′) that contain the NcoI and SmaI restriction sites, respectively. All the plasmid constructs were confirmed by sequencing.

### 4.3. β-Galactosidase Assays

β-Galactosidase activity was routinely assayed on log-phase bacterial cultures, as described by Miller [69].

### 4.4. Determination of Minimal Inhibitory Concentrations (MIC)

MIC values of antibiotics were determined by the microdilution method in Mueller Hinton II broth (MHIIB) (Sigma). Susceptibilities were determined in 96-well microtiter plates with an inoculum of 2 × 10^5^ cfu in 200 µL containing two-fold serial dilutions of each compound. The MIC was defined as the lowest concentration of each compound for which no visible growth was observed after 18 h of incubation at 37 °C. Each assay was systematically performed in triplicate. The average of three independent assays was considered in μg/mL.

### 4.5. Preparation of the Microtiter Plates for β-Galactosidase Assays

The standard β-galactosidase assay was adapted for compound screening by using 96-well microtiter plates and a SUNRISE^TM^ Tecan for absorbance readings. Briefly, strains were grown to an OD_600_ of 0.6. Cultures were diluted to an OD_600_ of 0.2, and added (200 µL) to the Phenotype MicroArrays ™ test plates (Biolog plates PM11 to PM19) (Supplementary Data 2). After overnight incubation at 37 °C, cells were centrifuged, washed, and treated with ONPG (2-nitrophenyl β-d-galactoside, Sigma) (4 mg/mL). Curves of OD_420_ were plotted over the time (30 min) to identify optimal inducers (Supplementary data 2). Similar experiments were repeated in 96-well microtiter plates preloaded with a chosen concentration range for each compound: each well was loaded with 20 μL of ONPG (4 mg/mL) and 10 μL of compound dilutions (Supplementary Data 3), then cells (170 µL at an OD_600_ of 0.2) were added. The plates were incubated at 37 °C inside the reader, and curves of OD_420_ were plotted over the time (6 h). The obtained readings in presence of ONPG were used to calculate Miller units and for determining the fold change in *lacZ* activity, relatively to standard growth conditions. Experiments were independently repeated at least three times.

### 4.6. Preparation of OM Extracts

Bacterial cultures (50 mL), grown in the presence or absence of stress, were incubated according to the optimum *micC/ompN* induction conditions determined by the β-galactosidase assay. The cells were washed and concentrated 12.5 fold in 20 mM sodium phosphate buffer (pH 7.4), and lysed by one passage through a cell disruptor (Constant Systems) at 2 kbar. After removal of cell debris by centrifugation (7000× *g*, 20 min, 4 °C) the supernatant was ultracentrifuged (100,000× *g*, 60 min, 4 °C) to collect the whole cell envelopes. These were resuspended in 0.3% *N*-laurylsarcosinate, and incubated for 30 min at room temperature to solubilize the IM. The insoluble OM extracts were pelleted by centrifugation (100,000× *g*, 60 min, 4 °C).

### 4.7. SDS-PAGE and Western Blot Analysis

OM were prepared as described above, resuspended in 20 mM sodium phosphate buffer (pH 7.4), and kept at −20 °C until use. All samples were diluted in Laemmli buffer (2×: 4% SDS, 20% glycerol, 10% 2-mercaptoethanol, 0.004% bromophenol blue, 125 mM Tris-HCl, pH 6.8) and heated for 5 min at 100 °C before loading. Samples corresponding to 0.2 OD units were separated on 10% SDS-PAGE. To better resolve OmpF and OmpC, 4 M urea was added to the running gel. Proteins were either visualized after straining with Coomassie Brilliant Blue R250 or transferred onto nitrocellulose blotting membranes (GE Healthcare, Aulnay-sous-Bois, France). Primary rabbit antibodies and dilutions were: TolC (1:5000), OmpFd (1:5000), OmpC1 (1:5000), and OmpN (1:1000). Goat anti-rabbit HRP-conjugated secondary antibodies and Clarity Max™ Western ECL Blotting substrates (Bio-Rad, Marnes-la-Coquette, France) were used for detection. Protein bands were visualized with a molecular imager Chemidoc-XRS System (Bio-Rad) and quantified using the Image Lab software (Bio-Rad) by using the TolC band as a standard. Peptide-specific antibodies were used to avoid cross-detection of OmpC and OmpN: OmpC1 antibodies are directed against KNGNPSGEGTSGVTNNG amino acid sequence present in loop 4 [70], and OmpN1 antibodies are directed against the GGADNPAGVDDKDLVKYAD amino acid sequence found in loop 7 (Thermo Scientific Pierce custom antibody service, Villebon-sur-Yvette, France).

### 4.8. Whole Cell-Based Viability Assay

Resazurin-based CellTiter-Blue^®^ Cell Viability Assay (Promega) was used to determine the metabolic inhibition of cells expressing single porins in the presence of clinically relevant antibiotics as an indicator of porin permeation properties [6]. These assays were performed on W3110 derivatives, i.e., W3110ΔF (expressing OmpC), W3110ΔC (expressing OmpF), and W3110ΔFC transformed with pTrc99A-*ompN* (expressing OmpN). Overnight cultures were diluted to 1:100 and grown until mid-log phase in MHIIB. Strain containing pTrc99A-*ompN* was grown in the presence of Amp, and OmpN expression was induced with 0.1 mM IPTG for 1 h at 37 °C. When tested for β-lactam permeation, cultures were diluted to 10^7^ cells/mL in fresh MHIIB containing 10% of CellTiter Viability Reagent. For strains containing pTrc99A-*ompN*, MHIIB was supplemented with 0.1 mM IPTG, and β-lactamase inhibitors tazobactam and clavulanic acid (4 µg/mL each), to inhibit the activity of the plasmidic AmpC, but not Amp. Microtiter plates (96 well) with black sides and a clear bottom were preloaded with 10 μL of 20× concentrated antibiotic solutions. For each antibiotic, the final concentration in the wells was defined as the maximal concentration that did not alter the metabolism of the porin-less strain, i.e., ertapenem, 0.125 µg/mL; meropenem, 0.125 µg/mL; cefotaxime, 0.0625 µg/mL. Cells (190 µL) were then added to separate wells. Control wells also contained cells with resazurin, but no antibiotic, and resaruzin with antibiotics without cells. Fluorescent signals of resorufin were measured with a TECAN Infinite Pro M200 spectrofluorometer (excitation wavelength 530 nm and emission wavelength 590 nm). Kinetic readings were taken at 37 °C every 10 min for 300 min. The % of metabolic inhibition for each strain exposed to each antibiotic was calculated from the measured difference of relative fluorescence units (RFUs) in the presence (RFU_ATB_) as compared to in the absence (RFU_MAX_) of antibiotic. All experiments were performed at least four times.

### 4.9. Colicin Killing Assays

LB agar plates were overlaid with 4 mL of soft agar (with a final agar concentration of 0.75%) containing 100 µL of *E. coli* overnight cultures. Serial two-fold dilutions of ColE2 or ColE3 (laboratory collection), were spotted in 5 µL drops onto the lawns, and the plates were incubated overnight at 37 °C. Efficiencies of killing were taken as the reciprocal of the highest dilution that gave complete clearing of the lawn.

## 5. Conclusions

Altogether, these data suggest that exposure to β-lactams induce a complex stress response to reduce the translocation of these antibiotics across the OM in *Enterobacteriaceae*. Further work will analyze how external stresses, such as β-lactams, interact with the σ^E^ envelope stress response and H-NS in laboratory strains, as well as in MDR clinical isolates.

## Figures and Tables

**Table 1:** Strains and plasmids used in this study.

Strain or Plasmid	Description	Source or Reference
*E. coli* strains		
MC4100	F^−^ *[araD139]_B/r_* Δ*(argF-lac)169 λ^−^ e14 flhD5301* Δ*(fruK-yeiR)725(fruA25) relA1 rpsL150*(Str^R^) *rbsR22* Δ*(fimB-fimE)632(::IS1) deoC1*	[60]
MH1160	MC4100 *ompR101*	[61]
TR49	MC4100 λRS88[*degP–lacZ*]	[47]
W3110	F^−^ *λ^−^ IN(rrnD-rrnE)1 rph-1*	[62]
SR8265	W3110 *rybB< >aph*, Kan^R^, source for P1 transduction	[63]
PS2209	W3110 Δ*lacZ169*	[64]
PS2652	Δ*lacZ169 zch-506::TnlO hns-1001::*Tn*seq1*, Kan^R^, source for P1 transduction	[64]
AG100	F^−^ *glnX44*(AS) *galK2*(Oc) *rpsL704*(Str^R^) *xylA5 mtl-1 argE3*(Oc) *thiE1 tfr-3*	[65]
CH164	AG100 *marA zdd-230*::Tn9, Cam^R^, source for P1 transduction	[66]
BW25113	F^−^ Δ*(araD–araB)567* Δ*lacZ4787*(::rrnB-3) *λ^−^ rph-1* Δ*(rhaD–rhaB)568 hsdR514*	[67]
JW4130	BW25113 *hfq*::*kan*, Kan^R^, source for P1 transduction	GE Healthcare
SD01	MC4100 Δ*rybB*	This study
SD02	MC4100 *marA zdd-230*::Tn9, Cam^R^,	This study
SD03	MC4100 Δ*hfq*	This study
SD04	MC4100 Δ*hns*	This study
SD05	MC4100 Δ*rpoS*	This study
W3110Δ*ompF*	W3110 *ompF::kan*	M.G. Page
W3110Δ*ompC*	W3110 *ompC::kan*	M.G. Page
W3110Δ*ompF*Δ*ompC*	W3110 Δ*ompF*Δ*ompC*	M.G. Page
Plasmids		
pDrive	PCR cloning vector; Amp^R^, Kan^R^	Qiagen
pRC1	pDrive containing *Enterobacter aerogenes* MarA	[31]
pRC2	pDrive containing *Enterobacter aerogenes* RamA	[32]
pSD01	pDrive encoding MicC sRNA	This study
pFus2K	Cloning vector with promoter-less *lacZ*, Kan^R^	[59]
pSD02	pFus2K containing the *micC–lacZ* fusion	This study
pSD03	pFus2K containing the *ompN–lacZ* fusion	This study
pTrc99A	Expression vector with the inducible P_TRC_ promoter, Amp^R^	Pharmacia
pSD04	pTrc99A containing OmpN	This study
pSD05	pTrc99A containing RseA	This study
pBAD24	Expression vector with the inducible P_BAD_ promoter, Amp^R^	[68]
pBAD24-NlpE	pBAD24 containing NlpE	M. Masi
pBAD33	Expression vector with the inducible P_BAD_ promoter, Cam^R^	[68]
pBAD33-CpxA*	pBAD33 containing an autoactivated (*) CpxA	M. Masi

## Supplementary Materials

The following are available online at www.mdpi.com/2079-6382/6/4/33/s1, Supplementary Data 1: Screening of *micC* expression by β-galactosidase assay using preloaded 96-well Phenotype MicroArrays^TM^ plates (Biolog PM11 to PM19) for bacterial chemical susceptibility, Supplementary Data 2: Fifteen compounds were selected to investigate their effects on MicC and OmpN, Supplementary Data 3: The expression of OmpN was evaluated in laboratory and clinical strains of *E. coli* by Western blot analysis, Supplementary Data 4: Partial *pfo*(*ybdK*)-*micC*-*ompN* genetic region.
