# Supplementary material for: Dual Regulation of the Small RNA MicC and the Quiescent Porin OmpN in Response to Antibiotic Stress in Escherichia coli
Source: Antibiotics (Basel). 2017 Dec 6;6(4):33. doi: 10.3390/antibiotics6040033 (PMC5745476; doi:10.3390/antibiotics6040033)
Supplement: Supplementary file 1 [file antibiotics-06-00033-s001.pdf]

antibiotics

MDPI

Dual regulation of the small RNA MicC and the quiescent porin OmpN in response to antibiotic stress in *Escherichia coli*

Supplementary Data 1. Screening of *micC* expression by β-galactosidase assay using preloaded 96-well Phenotype MicroArrays™ plates (Biolog PM11 to PM19) for bacterial chemical susceptibility. Two technical replicates were assayed, and the Miller units for each compound were calculated at 30 minutes. The fold activity was calculated based on the Miller units obtained from cells grown standard conditions, in the absence of inducer.

	PM11C	PM12B	
Amikacin			Penicillin G
Chlortetracycline			Tetracycline
Lincomycin			Carbenicillin
Amoxicillin			Oxacillin
Cloxacillin			Penimepicycline
Lomefloxacin			Polymyxin B
Bleomycin			Paromomycin
Colistin			Vancomycin
Minocycline			D,L-Serine hydroxamate
Capreomycin			Sisomicin
Demeclocycline			Sulfamethazine
Nafcillin			Novobiocin
Cefazolin			2,4-Diamino-6,7- diisopropylpteridine
Enoxacin			Sulfadiazine
Nalidixic acid			Benzethonium chloride
Chloramphenicol			Tobramycin
Erythromycin			Sulfathiazole
Neomycin			5-Fluoroorotic acid
Ceftriaxone			Spectinomycin
Gentamicin			Sulfamethoxazole
Potassium tellurite			L-Aspartic-β- hydroxamate
Cephalothin			Spiramycin
Kanamycin			Rifampicin
Ofloxacin			Dodecyltrimethyl ammonium bromide

	PM13B	PM14A	
Ampicillin			Acriflavine
Dequalinium chloride			Furaltadone
Nickel chloride			Sanguinarine
Azlocillin			9-Aminoacridine
2, 2'-Dipyridyl			Fusaric acid
Oxolinic acid			Sodium arsenate
6-Mercaptopurine			Boric Acid
Doxycycline			1-Hydroxypyridine -2- thione
Potassium chromate			Sodium cyanate
Cefuroxime			Cadmium chloride
5-Fluorouracil			Iodoacetate
Rolitetracycline			Sodium dichromate
Cytosine-1-beta D-arabinofuranoside			Cefoxitin
Geneticin (G418)			Nitrofurantoin
Ruthenium red			Sodium metaborate
Cesium chloride			Chloramphenicol
Glycine			Piperacillin
Thallium (I) acetate			Sodium metavanadate
Cobalt chloride			Chelerythrine
Manganese chloride			Carbenicillin
Trifluoperazine			Sodium nitrite
Cupric chloride			EGTA
Moxalactam			Promethazine
Tylosin			Sodium orthovanadate

	PM15B	PM16A	
Procaine			Cefotaxime
Guanidine hydrochloride			Phosphomycin
Cefmetazole			5-Chloro-7-iodo- 8-hydroxyquinoline
D-Cycloserine			Norfloxacin
EDTA			Sulfanilamide
5,7-Dichloro- 8- hydroxyquinaldine			Trimethoprim
5,7-Dichloro-8- hydroxyquinoline			Dichlofluanid
Fusidic acid			Protamine sulfate
1,10- Phenanthroline			Cetylpyridinium chloride
Phleomycin			1-Chloro -2,4- dinitrobenzene
Domiphen bromide			Diamide
Nordihydroguaia retic acid			Cinoxacin
Alexidine			Streptomycin
5-Nitro-2- furaldehyde semicarbazone			5-Azacytidine
Methyl viologen			Rifamycin SV
3, 4-Dimethoxybenzyl alcohol			Potassium tellurite
Oleandomycin			Sodium selenite
Puromycin			Aluminum sulfate
CCCP			Chromium chloride
Sodium azide			Ferric chloride
Menadione			L-Glutamic-ghydroxamate
2-Nitroimidazole			Glycine hydroxamate
Hydroxyurea			Chloroxylenol
Zinc chloride			Sorbic acid

	PM17A	PM18C	
D-Serine			Ketoprofen
β-ChloroL-alanine hydrochloride			Sodium pyrophosphate decahydrate
Thiosalicylic acid			Thiamphenicol
Sodium salicylate			Trifluorothymidin
Hygromycin B			Pipemidic Acid
Ethionamide			Azathioprine
4-Aminopyridine			Poly-L-lysine
Sulfachloropyridazine			Sulfisoxazole
Sulfamonomethoxine			Pentachlorophenol
Oxycarboxin			Sodium m-arsenite
3-Amino-1,2,4- triazole			Sodium bromate
Chlorpromazine			Lidocaine
Niaproof			Sodium metasilicate
Compound 48/80			Sodium m-periodate
Sodium tungstate			Antimony (III) chloride
Lithium chloride			Semicarbazide
DL-Methionine hydroxamate			Tinidazole
Tannic acid			Aztreonam
Chlorambucil			Triclosan
Cefamandole nafate			3,5-Diamino- 1,2,4-triazole (Guanazole)
Cefoperazone			Myricetin
Cefsulodin			5-fluoro-5'- deoxyuridine
Caffeine			2-Phenylphenol
Phenylarsine oxide			Plumbagin

	PM19B	PM20B	
Josamycin	1-3 fold	6-10 fold	Amitriptyline
Gallic acid	1-3 fold	1-3 fold	Apramycin
Coumarin	1-3 fold	1-3 fold	Benserazide
Methyltrioctylammonium chloride	> 10 fold	> 10 fold	Orphenadrine
Harmane	1-3 fold	1-3 fold	D,L-Propranolol
2,4-Dintrophenol	1-3 fold	1-3 fold	Tetrazolium violet
Chlorhexidine	1-3 fold	> 10 fold	Thioridazine
Umbelliferone	1-3 fold	3-6 fold	Atropine
Cinnamic acid	1-3 fold	1-3 fold	Ornidazole
Disulphiram	1-3 fold	1-3 fold	Proflavine
Iodonitro Tetrazolium Violet	1-3 fold	1-3 fold	Ciprofloxacin
Phenyl- methylsulfonylfluoride (PMSF)	1-3 fold	1-3 fold	18-Crown-6 ether
FCCP	6-10 fold	6-10 fold	Crystal violet
D,L-Thioctic Acid	1-3 fold	1-3 fold	Dodine
Lawsone	1-3 fold	6-10 fold	Hexachlorophene
Phenethicillin	3-6 fold	1-3 fold	4-Hydroxycoumarin
Blasticidin S	1-3 fold	1-3 fold	Oxytetracycline
Sodium caprylate	3-6 fold	> 10 fold	Pridinol
Lauryl sulfobetaine	> 10 fold	1-3 fold	Captan
Dihydrostreptomycin	1-3 fold	1-3 fold	3,5-Dinitrobenzene
Hydroxylamine	1-3 fold	1-3 fold	8-Hydroxyquinoline
Hexammine cobalt (III) chloride	1-3 fold	1-3 fold	Patulin
Thioglycerol	1-3 fold	1-3 fold	Tolylfluanid
Polymyxin B	6-10 fold	1-3 fold	Troleandomycin

(red)	> 10 fold
(orange)	6-10 fold
(amber)	3-6 fold
(yellow)	1-3 fold

Supplementary Data 2. Fifteen compounds were selected to investigate their effects on MicC and OmpN. 5 (nordihydroguaiaretic acid, thioridazine HCl, benzethonium chloride, promethezium HCl, colistin, and chlorpromazine HCl) of them were identified as inducers of the micC– and ompN–lacZ fusions by using Phenotype Microarrays plates (Biolog PM11 to PM19). First, minimal inhibitory concentrations (MICs, μg/ml) were determined as described in the Materials and Methods section. Range of concentrations for each compound was then chosen according to the MIC for the measurements of β-galactosidase activities by using the microtiter plate method. The final working concentrations were chosen as the lowest concentrations that produced the maximal changes to the β-galactosidase activities.

Compound	MIC (μg/ml)	Range of concentration tested (μg/ml)	Chosen concentration (μg/ml)
Benzalkonium chloride	0.2	0.1-0.8	0.4
Nordihydroguaiaretic acid	100	50-400	200
Doripenem	0.2	0.1-0.8	0.4
Thioridazine HCl	100	50-400	200
Benzethonium chloride	100	50-400	200
Meropenem	1	0.5-8	2
Imipenem	1.25	0.3125-10	1.25
Promethezium HCl	200	200-1600	800
Biapenem	0.32	0.8-3.2	3.2
Polymyxin B	1	1-16	2
Colistin	1	1-16	2
Ceftazidime	1	1-16	4
Cefepime	1	1-16	2
Ertapenem	1	1-16	6
Chlorpromazine HCl	200	200-1600	400

Supplementary Data 3. The expression of OmpN was evaluated in laboratory and clinical strains of *E. coli* by Western blot analysis. Briefly, cells were grown overnight in LB broth, spun down, resuspended in 1× Laemmli buffer. Samples were heated for 5 min at 95 °C prior to protein separation by SDS-PAGE and electrotransferred onto nitrocellulose blotting membranes. The strains were selected based on their OmpF and OmpC expression profile, as well as their resistance towards β-lactam (CAZ, ceftazidime and ERT, ertapenem) and other (CM, chloramphenicol). These data are from Pagès *et al.* [2].

***E. coli* strains**	**Porin expression**			**MIC (µg/ml)**			**β-lactamases**
	OmpF	OmpC	OmpN	CAZ	ERT	CM	AmpC
AG100	+	+	-	0.5	0.02	0.125	AmpC
AG100A	+	+	-	0.5	0.02	0.031	AmpC
AG100Atet	+	+	-	1	0.02	0.5	AmpC
ARS100	-	-	-	1	2	32	AmpC
ARS108	-	-	-	32	4	>128	CTX-M-15
ARS144	+	+	-	1024	0.5	>128	CTX-M-15, DHA-1
ARS150	+	+	-	512	2	>128	CTX-M-15, TEM-1
ARS183	+	+	-	64	1	>128	CTX-M-14, TEM-1
ARS237	+	+	-	128	4	>128	CTX-M-14, TEM-1
ARS273	-	+	-	128	8	>128	CTX-M-15

+: presence; -: absence

Supplementary Data 4. Partial *pfo*(*ybdK*)-*micC*-*ompN* genetic region. *micC* (in green) is transcribed clockwise on the *E. coli* chromosome on the opposite strand of the adjacent *ompN* and *pfo* (former *ybdK*) genes (in blue). Relevant characteristics of the region are indicated, such as the *pfo* stop codon (blue star) and the *ompN* start codon (blue, +1). Primer extension analysis of MicC RNA [1] allowed the precise position of the MicC transcriptional start (green, +1), −10 and −35 promoter sequences (green, underlined). The position of the *ompN* −10 and −35 promoter sequences was predicted by using the BPROM software, available at http://www.softberry.com/berry.phtml?topic=bprom. Positions and sequences of the primers used for PCR-amplifying fragments of this region are indicated by arrows.

```
                                                         SD5
AATTCGCAGCAGCCAGAAGTGGCAGAACAGTTATGGAAAGATGCTGCAGCTGATTTGCAAAAACGCTATGACTTC
                              Pfo(YbdK)→      *
CTGGCACAAATGGCCGGAAAAGCGGAAAAAAAGCAACACCGATTAAATGCTCTGGATAAGGATTATCCAATTCTAA

AAAAAAAGCCCGGACGACTGTTCGGGCTTGTCTTTTTATATGTTGGAAAATCAGTGGCAATGCAATGGCCCAACA
                          MicC ← +1        SD2  SD4
GAAAATAAAATCTGTGACAATAAAGGCATATAACCCGCGCAGAATAACGTATAGCAAATTAAAAGTATAATTTTT
                                            -10                     -35
ATTTTGTATGTATTATCTTCGATACATTCACCAGACTTATTATCATTTCAGAGGAATTATTTGATTAAGGTTTTA
                             -35                          -10
CTTAAGGCGTAACAAATGATATTTATCGGCTAACTGAACTTCTCCTTTAGGATGTTTTCACTCCCCGAAATGGGG
                                                               SD1
                                       +1 → OmpN
ATAAAAGGCAAATAAAATAACTAAAGGATTTATTCAATGAAAAGCAAAGTACTGGCACTTTTAATTCCTGCCCTG
                         SD3
```

References

1. Chen, S.; Zhang, A.; Blyn, L.B.; Storz, G. MicC, a second small-RNA regulator of Omp protein expression in Escherichia coli. *J. Bacteriol.* **2004**, 186, 6689–6697.
2. Pagès, J.M.; Peslier, S.; Keating, T.A.; Lavigne, J.P.; Nichols, W.W. Role of the outer membrane and porins in susceptibility of β-lactamase-producing *Enterobacteriaceae* to ceftazidime-avibactam. *Antimicrob. Agents Chemother.* **2015**, 60, 1349–1359.

© 2017 by the authors. Submitted for possible open access publication under the terms and conditions of the Creative Commons Attribution (CC BY) license (http://creativecommons.org/licenses/by/4.0/).
